# Behavioural Phenotypic Plasticity of Submerged Oviposition in Damselflies (Insecta: Odonata)

**DOI:** 10.3390/insects10050124

**Published:** 2019-04-29

**Authors:** Jana Branwen Helebrandová, Petr Pyszko, Aleš Dolný

**Affiliations:** 1 Department of Biology and Ecology, Faculty of Science, University of Ostrava, 710 00 Ostrava, Czech Republic; xaustrik@seznam.cz (J.B.H.); pyszko.petr@gmail.com (P.P.); 2 Faculty of Science, Institute of Environmental Technologies, University of Ostrava, 710 00 Ostrava, Czech Republic

**Keywords:** adaptive learning, adult experience, *Lestes sponsa*, manipulative experiment, sex-specific difference, oviposition tactic, reproductive behaviour

## Abstract

Emerald damselfly *Lestes sponsa* is a common species within the temperate zone, with no special need for protection. The tactic of submerged oviposition is well known from other Odonata species, but has rarely been noticed or described in *Lestes sponsa*. Our study investigated the tactics of oviposition in this species, and shows that submerged oviposition indeed occurs frequently in *Lestes sponsa*. We experimentally tested the difference in the roles of males and females during the submerged ovipositional behaviour by combining males/females from submerging populations with males/females from non-submerging populations. We discovered that, whereas submerging males coupling with non-submerging females did not lead to submersion, the opposite combination of pairs submerged. Other patterns of submersions are discussed further in this paper. Our research led to the conclusion that damselflies have the ability to learn and react to different situations in keeping with the learning potential of insects in general.

## 1. Introduction

Successful production of viable offspring is the main life goal of every animal. The Odonata have many strategies and tactics that lead to greater competitiveness among adults or their offspring [1], and submerged oviposition is one of them. Whilst endophytic oviposition is typical for Zygoptera, and also for the dragonfly family Aeshnidae [1], in the submerged type of endophytic oviposition, females lay their eggs into plant tissue beneath the water.

The main advantage of submerged oviposition for adult females is the avoidance of male harassment; for adult males, it is the minimizing of competition with other males [2]. Adult selection of oviposition sites, including preferences for submerged sites, can positively affect embryonic development and hatching success of their eggs, as well as larval development [3,4]. Underwater position of eggs can also provide protection from egg parasitoids [5], which even applies when submerged plants are utilized as oviposition substrates [6]. There are different variants of submerged oviposition tactic. For example, in *Calopteryx cornelia* [7], females oviposit underwater while males non-contact guard them above the water level. Males of *Enallagma hageni* rescue their females after improper resurfacing [8], but they never go under the water, similarly for *Enallagma cyathigerum* [9]. In contrast, in *Lestes sponsa* (Hansemann, 1823) (Odonata: Lestidae), tandem guarding results in both female and male submerging together [2,9,10]. However, in other submerging species of Odonata, the roles of male and female during oviposition vary a lot. Even in visibly connected tandems of *Lestes sponsa* the male and female roles are different.

The tactic of submerged oviposition is commonly found in some species of the damselfly families Coenagrionidae and Calopterygidae [7,11,12,13,14]; however, in the Lestidae it is rare, with only a few reports dealing with submerged oviposition, generally focused on just a few specimens or occasionally couples with no further details provided [2,9,15,16,17]. While it appears that all species of the family Lestidae generally prefer oviposition above the water surface, with the frequency of underwater oviposition relatively low on a regional scale, some recent findings have led to the conclusion that under certain conditions at a micro- or mesoscale, the phenomenon of the submerged oviposition may become a common tactic [10,18]. These results suggest that populations within the same species have different ovipositional histories. In the case of *Lestes* sponsa, submerged oviposition is particularly noticeable at regional and local scales, in contrast, for example, to *Erythromma lindenii*, in which it is found among geographically separated populations across the whole distribution area of the species [14].

The choice of water site, plant species and its position, and the timing of submerged ovipositing, are necessary for successful breeding and ovipositing. Correct assessment of these factors seems to come with experience and involves associative learning, about what triggers different reactions to varying situations as a result of life experiences [19]. Associative learning has been proven in many insect orders [20]. Most studies are focused on Hymenoptera [21,22,23,24,25], but there is already published research on associative learning in Odonata, in the Aeshnidae [26] and especially in the Coenagrionidae [27,28,29], which is the family in which submerged oviposition has been studied [8,30,31,32,33]. Most studies in the field of associative learning in the order Odonata have only focused on larvae, chemical stimuli and chemosensory recognition in water [27,28]. To the authors’ best knowledge, very few publications discuss the issue of behavioural changes in odonates as a consequence of experience, and apart from the study by Miller and Fincker [29], there is a lack of research addressing changes in behaviour of adults as a result of practice.

Surveys of oviposition behaviour in damselflies, such as the one conducted by Helebrandová et al. [18], have reported the potential of manipulative approaches for the investigation of these issues. The authors demonstrated that a carefully designed net cage with semi-natural conditions provided behavioural results comparable to natural stands. The prospect of being able to research under conditions that can be manipulated serves as an incentive to spur on our recent research.

To better understand the cues and restraints of submerged oviposition, particularly the plasticity of this behaviour, we performed a manipulative experiment on the ovipositional behaviour of *L. sponsa* using a net cage with both inexperienced (naive) and experienced adult specimens. Here, we used *Lestes sponsa* as a representative damselfly species which employs a facultative submerged oviposition tactic in response to particular features of the environment and/or individuals’ experience. Our two original hypotheses were:

(1) The roles of males and females differ during oviposition; every specimen can influence the process individually. Females are more active than males in terms of ovipositional behaviour. The difference can be especially noticeable when a specimen from a non-submerging site interacts with a specimen from a submerging population. In sum, the females have dominant roles for the occurrence of submerging behaviour.
(2) Inexperienced (naive) damselflies are not fixed in one mode of ovipositional behaviour (above water), and their behaviour is not determined solely by inborn heredity. Even inexperienced specimens can change a behavioural pattern and can oviposit underwater under changed or different environmental conditions. The opportunities to interact with experienced individuals are of great importance in this context and we expect that inexperienced damselflies can oviposit underwater only if they interact with experienced individuals. Conversely, when conditions change, submerging will not be realised by the experienced damselflies either.

## 2. Materials and Methods

### 2.1. Source Sites

Populations of *Lestes sponsa* in all source sites had been established for more than ten years, and all sites were observed for at least two seasons. All populations with submerged oviposition (SUB) reached a similar size each year, with at least 500 specimens (with submerging during the summer season as a common tactic); the population that did not submerge (NON-SUB) was smaller, with between 100 and 200 specimens. The average distance between two locations was 18.5 km, the smallest 5.6 km, the longest 33 km. Mixing of specimens from individual sites with respect to the dispersing ability of *Lestes sponsa* [34] was practically impossible.

#### 2.1.1. Populations with Submerged Oviposition

• Štramberk Botanical Garden (SUB-1)

The former limestone quarry is situated near the town of Štramberk, in the north-east of the Czech Republic (49°35′19″ N, 18°7′30″ E). At the bottom of the quarry, there are clean shallow pools with a wetland character, where *Lestes sponsa* generally prefers to oviposit underwater [10]. Typical plants used for submerged oviposition are *Eleocharis palustris*, *Schoenoplectus lacustris* or even *Equisetum variegatum*. No fish are present in the water, which is therefore highly transparent.

• Borovec Pond (SUB-2)

The fish pond for rearing the young of the common nase fish (*Chondrostoma nasus*), of size 0.8–8 cm, is located near Příbor, in the north-east of the Czech Republic (49°38′7″ N, 18°6′4″ E). The plant used here was *Eleocharis palustris*. The water is clean, with the fish population not affecting its transparency. 

• Vítkov Lake (SUB-3)

The protected system of lakes near Vítkov, in the north-east of the Czech Republic, (49°46′33″ N, 17°46′19″ E) hosts not just damselflies, but many amphibians as well. The water is shallow and transparent, and *Lestes sponsa* uses *Juncus effusus* and *Schoenoplectus lacustris* to submerge.

#### 2.1.2. Non-Submerging Population

• Studénka Pond (NON-SUB)

The site is situated near Studénka in the north-east of the Czech Republic (49°41′58″ N, 18°4′27″ E). It is part of a pond system for keeping fish, water transparency is low and no typical preferred plant species (e.g., *Eleocharis palustris*, *Schoenoplectus lacustris*) are present here. Damselflies, which originated here, are called “naive” because they have never submerged under the water when ovipositing, nor had any previous generation on Studénka site over the previous ten years. 

### 2.2. Experimental Site

During the 2014 and 2015 seasons, conditions for the damselfly net cage were prepared in the Botanical Garden of the University of Ostrava (49°49′39.268″ N, 18°19′31.635″ E) such that the population of *Lestes sponsa* could be studied there under semi-natural conditions suitable for manipulative experiments [18]. The net cage construction was completed in May 2016, creating one large space with a total area of 22 m^2^ across nine water tanks (six of them with a size of 70 × 40 × 50 cm, three of them with a size of 60 × 30 × 40 cm) containing selected plant species (*Alisma plantago-aquatica, Eleocharis palustris, Equisetum variegatum, Juncus effusus, Schoenoplectus lacustris* and *Typha latifolia*); the net cage was designed as a long-term solution, to get as close to natural conditions as possible. Each of the nine same-size water tanks had developed individually since 2014, so the water in each had a similar depth, transparency, hardness and pH, but each of them provided a different ecological microhabitat (presence/absence of algae, dystrophic/eutrophic character, character of plant growth). Owing to its design and carefully chosen materials, the cage was exposed to all kinds of weather, and provided the damselflies with undisturbed living conditions comparable with the natural conditions observed in the field [18].

### 2.3. Net Cage Experiments

The first specimens of *Lestes sponsa*, a pilot group of 30 adults (15 males and 15 females), were introduced to the caged area in 2015 from SUB-1; to support the initial population, a further 120 larvae were transferred from SUB-1 in April 2016.

At the beginning of August 2016, 15 adults (8 males and 7 females) from SUB-2 and 25 adults (13 males and 12 females) from SUB-3 were marked by permanent marker and transferred to the net cage using a moving box (30 × 30 × 20 cm, 30 air vents, kept in the shade) to compare the behaviour of the different gene pools. Additional *L. sponsa* damselflies of the different populations were marked and shifted to the cage at the beginning of August 2017; in addition to the original population from the previous years, the cage was occupied by another 15 specimens from SUB-2 (for comparison in that year, using the same sex ratio as before) and 40 specimens from NON-SUB (20 males and 20 females).

During both 2016 and 2017, the basic parameters of all submersions were measured (day of season, depth of submersion, time spent underwater, daytime hours and plant species used). Every tandem was observed very carefully while ovipositing to summarise general ovipositing features, and especially the features of the submerged oviposition tactic (which partner led the phases of submersion, oviposition, emersion or flight; which other factors influenced oviposition, e.g., weather, presence of other damselflies), as well as any possible abnormalities (e.g., aggressive behaviour, netting used as an ovipositional site). In every tandem, the origin of both male and female was noted too. Observations were made on every day with suitable weather between the beginning of July and mid-September in 2016 and 2017; all submersions were measured between 20th July and 9th September.

### 2.4. Data Analysis

To test our main hypothesis, we created a set of binomial generalised linear models with a logit link, to explain the presence/absence of submersions for particular pairs by major treatment (i.e., if the pairs belonged to an experiment with two submerging populations or with one submerging population and one non-submerging), and with the second explanatory variable of (a) female origin, (b) male origin, (c) particular combination of partners and (d) combination within or between the same population. Variables were either as simple effects or interactions with one of the other explanatory variables. In addition, individual models with only one explanatory variable and also a null model were created. Ultimately, the model selection was based on the Akaike information criterion (AIC).

Data were analysed and plots were created using the packages coin [35], MuMIn [36], car [37], lme4 [38] and sciplot [39] in R 3.4.1. software [40]. Original data from research are published as Supplementary Materials (Table S1) after acceptance of the manuscript.

## 3. Results

### 3.1. The Process of Submerged Oviposition and Male and Female Roles during the Interaction

**Figure 1:**
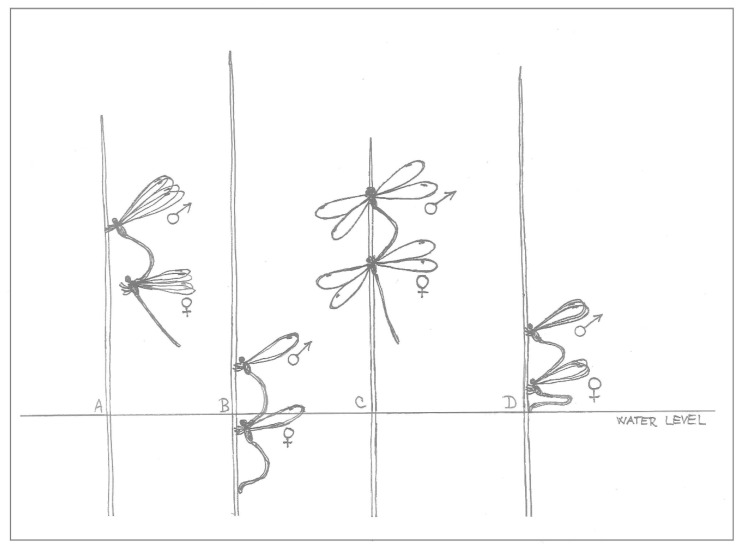
(**A**) Tandem of *Lestes sponsa* landing on the plant stem, male first; (**B**) ovipositing tandem with the female almost under the water; (**C**) the tandem drying their wings after final oviposition on the plant; (**D**) the female shaping her abdomen to refuse submersion.

The tandem was established by the male’s choice and action; once he had caught his chosen female behind her head, it was her turn to finish the copulatory position, the mating wheel. The oviposition itself was then started by the male leading the flight towards a suitable plant; he also landed on the stem first (Figure 1A). The female followed his movement during flight and landing, then she took control of the oviposition itself by pulling or pushing her connected male, including any possible submerging.

**Figure 2:**
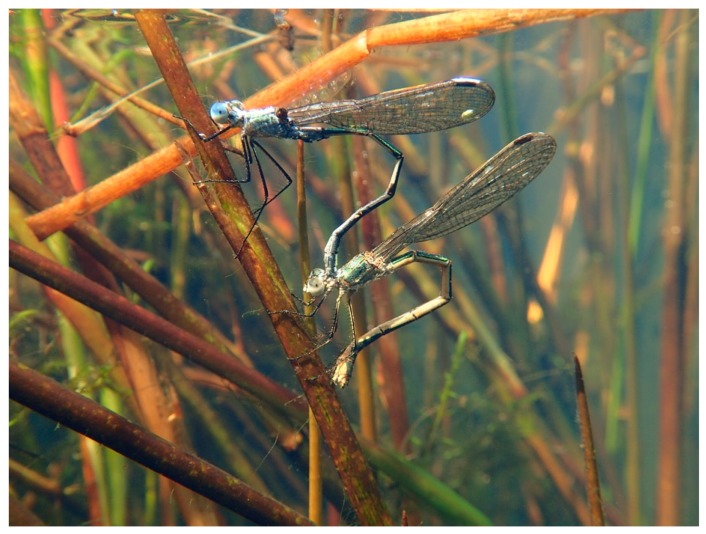
Tandem of *Lestes sponsa* ovipositing underwater in the net cage.

While the female climbed down the stem, pulling the male with her, she was mostly (especially at the beginning of the day) laying eggs into the plant tissue above the water, until she touched the water level with her abdomen. Then, she decided when to submerge; any inappropriate conditions (water less than 18 °C, strong wind or generally cold weather, presence of water striders, her individual inability to submerge) could make her interrupt ovipositing underwater for the moment. When she decided to submerge, she pulled the male with her under the water in her own time; even half-submerged, she could continue ovipositing (Figure 1B), and frequently the female would already be under the water with the male still above for a minute or more, until she moved down and the male was also submerged. During submersion, she moved according to the necessities of the egg laying process; she climbed down and around the plant stem freely, with the male following (Figure 2).

**Figure 3:**
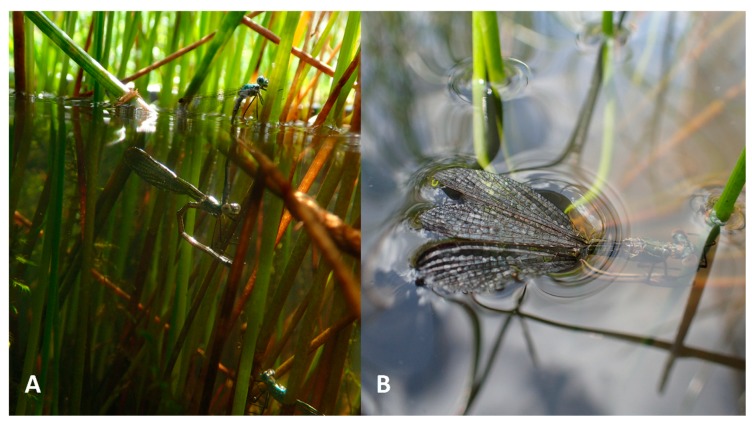
(**A**) The *Lestes sponsa* female continues to oviposit under the water while the male replenishes oxygen levels above the water; (**B**) a female from SUB-3 oviposits underwater with the top of her wings above the water.

The female also decided when to emerge for oxygen (short-term emergence before a subsequent submersion), sometimes with the female staying under the water to lay more eggs while she was still connected to the male, when their connection seemed to provide oxygen to her (Figure 3A). To finish the oviposition completely, she pushed the male out of the water.

After the final submersion on a particular plant, the tandem could continue ovipositing elsewhere. They dried their wings first by spreading them to the sides (Figure 1C). Then the female took off first, followed by the male, but during their time in the air, he took the lead again. A second option was when the particular tandem finished mating, mostly during late afternoon, but sometimes even in the middle of the day after just one oviposition (observed in 4.7% of all tandems). In these cases, the male released his grip of the female on the last used plant; he flew away from the plant, most frequently while the female was cleaning her head (especially her eyes), legs and abdomen. According to the time of day, the male could search for another partner, and the original female could be caught by another male, but, although some submersions were realised after 3:00 pm (Central European Summer Time), no new tandems were made after that time.

### 3.2. Interactions between the Experienced and Naive Damselflies

In general, the behaviour of all damselflies that had been removed from submerged-ovipositional localities in 2015–17 had similar character. On their first day in the net cage, in any year, they joined the already established net cage population in their activities, including mating and even ovipositing underwater. No abnormal competitive behaviour was observed, and there were no significant preferences for mating with partners from the same population when multiple submerged population gene pools were combined (d*f* = 1, χ^2^ = 1.281, adjusted *p* = 0.258).

In 2017, naive males and females from NON-SUB differed by their behaviour—they oviposited on the grass stems in the cage as frequently as on the water plants (damselflies from submerging populations have never been observed using grass for oviposition), and they even tried to use the grey plastic netting of the cage to oviposit. Tandems from NON-SUB frequently changed the plants for oviposition (their maximum was only a few minutes on one plant), compared with tandems made from members of the original submerging populations, which used a single plant stem for longer (mostly 20–40 min), as in the field. The NON-SUB tandems also used the whole length of plants, including the top, whereas submerging tandems only used approximately the 10 cm of stem above the water level.

Initially, the formation of mixed tandems of NON-SUB damselflies with net cage submerging damselflies was rather aggressive—previously established net cage tandems defended themselves by movements of their wings to avoid single male harassment, which was never seen in the net cage before. In one instance, a NON-SUB male was observed to catch a net cage female and eat her head instead of mating. The tendency to avoid mating between submerging and non-submerging specimens was almost significant (d*f* = 1, χ^2^ = 4.723, adj. *p* = 0.060). After three days, mixed tandems from SUB and NON-SUB specimens started to appear and engage in oviposition.

**Figure 4:**
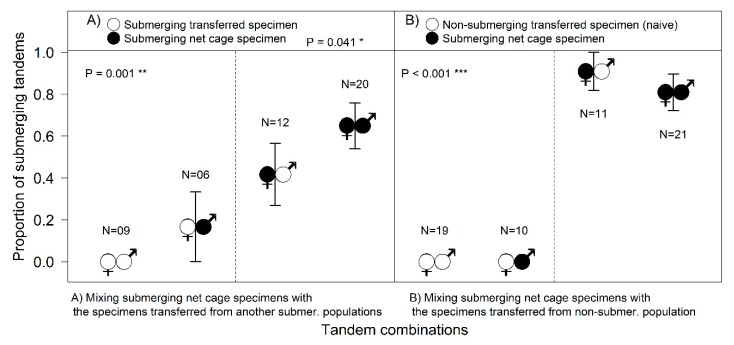
The proportion of submerging tandems in relation to the partner’s origin: (**A**) Net cage submerging specimens combined with submerging-experienced specimens from another submerging locality; (**B**) net cage submerging specimens combined with naive specimens from non-submerging locality. The female’s origin is crucial for the occurrence of submerging behaviour. Net cage females had greater proportion of submerged oviposition when combined with naive males than with transferred submerging males (d*f* = 104, χ^2^ = 4.19, *p* = 0.041). Net cage females had much greater proportion of submerged oviposition regardless of the male origin (d*f* = 104, χ^2^ = 64.04, *p* < 0.001) than the transferred females. This pattern was valid also for Figure 4A,B, tested separately (d*f* = 43, χ^2^ = 15.82, *p* = 0.001; d*f* = 57, χ^2^ = 56.61, *p* < 0.001, respectively). All analyses were performed by binomial GLM.

Based on AIC, as the selected best fitting model shows, the proportion of submerging tandems was surprisingly significantly higher if the submerging population was mixed with a non-submerging population (d*f* = 104, χ^2^ = 4.26, *p* = 0.039); the best explanatory factor was female origin (d*f* = 104, χ^2^ = 64.04, *p* < 0.001) and there was also significant interaction between female origin and the type of population mix (d*f* = 104, χ^2^ = 4.19, *p* = 0.041) (Figure 4).

Tandems with a newly transferred naive female did not start with underwater oviposition, with the few attempts by experienced males to push a naive female into the water not resulting in the females submerging (Figure 1D). Experienced females from the SUB-sites did not start submerging until they had spent some time in the cage (from a few days, to more than a week in 2015), so their contribution to the total number of submersions was not very high (Figure 4A). Experienced females submerged regardless of the male’s origin; females “taught” naive inexperienced males to submerge (Figure 4B), and the number of tandems laying their eggs underwater was surprisingly significantly higher with naive males than with males from another submerging population (Fisher’s Exact Test, *p* = 0.028).

Every year, a few specimens were killed and eaten by wasp spiders (*Argiope bruennichi*) and in particular by water striders (*Gerris lacustris*). Whereas wasp spiders catch anything, with no preference for damselflies, the approach of a water strider was the main reason for tandems already touching the water level to break off their oviposition; on the first or second occasions, they merely climbed higher up the stem, but if the attack was repeated more times, the tandem left the stem (or even the pool) to oviposit elsewhere. More difficulties appeared with tandems emerging from underwater: having no defence other than to emerge as quickly as possible, some damselflies became prey, mostly females because of their lower tandem position, especially in pools with higher water strider populations, where water striders were able to hunt in groups and waited for the submerging and emerging tandems hidden behind stems. Nevertheless, such deaths were observed only three or four times per season; the remainder of the water strider attacks only caused disturbances to the ovipositing damselflies.

## 4. Discussion

The tests revealed that the behavioural decision to submerge for oviposition differs in male and female damselflies. After transportation, specimens did not start submerging immediately (it could take them even one week), regardless of their population’s prior behaviour. Subsequently, the differences between males and females became obvious (Figure 4). Surprisingly, even naive males from non-submerging localities were able to contribute to the submersions under the leadership of an experienced female. Females transported from the submerging localities finally started to submerge in the net cage, but it took a while for them to join the process, so their contribution was not very high (Figure 4). The most striking result to emerge from the data was that naive females (the new comers from the non-submerging site) could not submerge even when paired with experienced males (Figure 1D and Figure 4).

The response of females appears to be hard-wired, at least for the adult stadium, but our research failed to give an explanation of its mechanism. Over time, it can be established in every population, but under non-submerging conditions, the population is adapted for above-water oviposition and it is advantageous to retain the behaviour without submerging to reduce general costs [19], even though the offspring would probably profit [3,5]. It may also be a response that develops during the larval stadium of females following assessment of the local environment. It is known from *Enallagma* species that damselfly larvae are able to learn and make decisions [27,28], so the possibility of the submerged ovipositional response being established during the larval period cannot be ignored; such a mechanism would provide the option for readaptation based on current conditions for every generation of damselflies independently, which would be very advantageous [20], especially when there is some plasticity for different situations. For example, females avoided submerging in pools with a high proportion of green algae, which could negatively affect the hatching of future larvae [13], but when the water there was cleaned, submerged ovipositions recommenced.

In comparison with females, who are more predictably successful, males have greater variation in reproductive success and they compete among each other to mate with multiple females (intrasexual selection). Sexual competition among males is connected with the existence of different behavioural patterns and tactics within a population, which is known from many damselfly species [13,41,42]. In this context, males of *Lestes sponsa* seem plastic: Even those who have never submerged before can do it under suitable environmental conditions and under the leadership of an experienced female. Males choose the plant for landing and ovipositing; plasticity of responses during mating is necessary within a mosaic of pools, as studied here in the net cage, and as manifested in natural conditions. Here, males of *L. sponsa* provide an example of plasticity of behaviour being advantageous [19].

Generally, we can say that the amount of experience grows during the mating period. During the second part of the ovipositional period, *Lestes sponsa* damselflies could go straight into the water, without previously ovipositing above water. They could also, after surfacing for oxygen, climb back down the stem to continue where they had left off, which suggests that a female can see her previous cuts on the stem, or can sense them mechanically; sometimes the female pulled the male under the water into a deeper position, without any apparent preceding ovipositions on the plant. These purely underwater ovipositions were often observed during the second half of August and beginning of September, in the afternoons, which suggests the damselflies would have some level of experience and memory (as observed already in larvae [27,28]). However, some tandems went deep on their first time (without any practice), which may indicate these two specimens are particularly compatible; the increased probability of repetitive submersion within these tandems was therefore not surprising. Conversely, some specimens broke their tandem after the first submersion without any outside reason (single male harassment, weather, etc.). Such behaviour reflects some individuality within *Lestes sponsa* species, which may influence their choices of partners and even special behaviour like the short solo submersions of single females (Figure 3B).

A possible explanation for identification of two different modes of oviposition behaviour in a particular insect species might be that this phenotypic plasticity in oviposition tactics can extend its habitat range. The present interpretation seems to be consistent with that of other research studies [3,4], which found that submerged oviposition has considerable benefits. The results obtained by Lambert et al. [3] suggest that submerged egg laying reduces costs associated with time stress and it leads to a higher fitness of offspring. From this perspective, the tactic of submerged oviposition can be considered as parental investment in the protection (increased survival) of offspring and an example of the trade-off between higher costs and risks for adults associated with particular facultative tactics and lower egg mortality as a result of parental investment in their protection [6]. However, this only applies to permanent waters and conversely the usability of temporary waters for the submerged oviposition is restricted by abiotic conditions and has limited benefits. With respect to the significantly different life history and developmental aspects of the species of the genus *Lestes* [4], we can assume that this tactic is advantageous only or predominantly for two (*L. sponsa* and *L. virens*) of all European species of the genus *Lestes*, including *Chalcolestes*.

Adaptive learning and plasticity are important in the life history of insects [20,43]. Our study has shown that behavioural plasticity, mediated partially through intraspecific communication, allows adaptation to novel conditions in new habitats, but it has also led to many questions. It would be interesting to assess if repeated experience with the submerged oviposition tactic leads to a higher frequency of the decision to submerge and/or adjust ovipositional tactic accordingly. Further research is required to confirm our findings, which suggest that sexual differences in preference for submerged ovipositing are especially strong under new conditions, such as an environmental change, and can be adaptive.

## 5. Conclusions

Our study has shown that the choices of individual damselflies have led to the evolution of a complex pattern of submerged oviposition behaviour, with roles during submergence varying between males and females. Furthermore, our research focusing on submerged oviposition has identified surprising facts about the skills and abilities of *Lestes sponsa*, revealing another example from the order Odonata of a wide range of behavioural modifications, with these damselflies having the capacity to react to different situations. The most obvious finding to emerge from this study was that specimens of different populations interact with each other, driving the resulting behaviours. This plasticity and adaptability is a keystone for the successful continuance of the prehistoric order of Odonata.

## Supplementary Materials

The following are available online at https://www.mdpi.com/2075-4450/10/5/124/s1, Table S1: Dataset for submerged oviposition experiment (with submerging and non-submerging population combinations).
